# Complete Genome Assembly and Annotation of Escherichia coli Bacteriophage 55 from Rivière la Quint in Gonaïves, Haiti

**DOI:** 10.1128/mra.00107-23

**Published:** 2023-06-05

**Authors:** Joshua C. Schwarz, William An, Austen Theroux, Luz D. Vargas, Benjamin K. Chan, Paul E. Turner, Alita R. Burmeister

**Affiliations:** a Department of Ecology and Evolutionary Biology, Yale University, New Haven, Connecticut, USA; b BEACON Center for the Study of Evolution in Action, East Lansing, Michigan, USA; c Program in Microbiology, Yale School of Medicine, New Haven, Connecticut, USA; d Center for Phage Biology and Therapy, Yale University, New Haven, Connecticut, USA; Loyola University Chicago

## Abstract

We present the structural and functional annotation of Escherichia coli bacteriophage 55, which has a genome length of 170,393 bp, with 219 predicted genes.

## ANNOUNCEMENT

Natural and contaminated habitats are potential sources for diverse bacteriophages, which are important sources for potential new bacteriophage-based therapies. Bacteriophage 55 was isolated from Rivière la Quint in Gonaïves, Haiti. The water sample was filtered through a 0.22-μm filter, followed by overnight enrichment at 37°C in tryptic soy broth on Escherichia coli strain BW25113. The enriched sample was centrifuged for 15 min at a relative centrifugal force (RCF) of 4,000, and the supernatant was filtered through a 0.22-μm syringe filter, followed by three rounds of isolation through single-plaque purification.

DNA was extracted using the Norgen phage DNA isolation kit according to the manufacturer’s instructions. DNA libraries were prepared and sequenced at MiGS (Pittsburgh, PA). Sample libraries were prepared using Nextera-based library preparation with IDT for Illumina 10-bp indices and were sequenced on an Illumina NextSeq 2000 system producing 2 × 151-bp reads, which resulted in 1,585,980 reads.

Genome assembly and annotation were performed using the Center for Phage Technology web-based programs Galaxy (1) and Apollo (2). All tools were run with default parameters unless otherwise specified. Sequences were analyzed for quality with FastQC (v.0.72+galaxy1) (3), trimmed with trimmomatic (Galaxy v.0.38.0) (4), and assembled with SPAdes (v.3.12.0+galaxy1) (5). The final assembly included a circular contig of 170,393 bp, with coverage of 216×. The 55-bp terminal overlap was manually trimmed. An NCBI BLASTn (6) search of the nucleotide collection within the standard database indicated that phage 55 is in the subfamily *Tevenvirinae* of the class *Caudoviricetes* and is closely related to Escherichia phage ST0 (GenBank accession number MF044457.1), with identity of 97.03% and query coverage of 96% (date of access, 22 September 2021). We reopened the assembly to be syntenic with the phage ST0 genome.

For structural annotation, we used the PAP structural workflow (v.2021.2) with algorithms GLIMMERr3 (v.0.2) (7), MetaGeneAnnotator (v.1.0.0) (8), and sixpack (v.5.0.0+galaxy2) (9) to predict gene locations. We manually assessed each putative gene location for Shine-Dalgarno sequences, start and stop codons, and the overlap or distance between genes.

For functional annotation, we used the Galaxy PAP Functional Workflow (v.2021.01) (10) to identify gene functions based on gene databases (Canonical Phages Database, SwissProt database, and NCBI nonredundant database [phages only]) and InterProScan (Galaxy v.5.52–86.0) (11). To confirm putative gene function, we manually analyzed the database predictions before finalizing the functional call. We used Artemis (v.18.2.0) (12) to confirm proper formatting of the entire annotation and to generate the sequin file for GenBank submission.

The final annotation contained 277 predicted genes, including 150 genes for hypothetical proteins, 127 genes for proteins with putative functions, and 3 tRNA genes. EDGE bioinformatics with gene family analysis active (13) predicted that the genome has a GC content of 37.65%, with no identified virulence factors.

### Data availability.

The genome of bacteriophage 55 has been added to NCBI GenBank under accession number OQ599310.1. Sequences are available in the Sequence Read Archive (SRA) under SRA accession number SRX19581772 and BioProject accession number PRJNA941886. For more robust identification, we also use RGB-004 as a unique internal identifier for the bacteriophage 55 isolate.
